# *Pseudomonas aeruginosa* Biofilm Lifecycle: Involvement of Mechanical Constraints and Timeline of Matrix Production

**DOI:** 10.3390/antibiotics13080688

**Published:** 2024-07-24

**Authors:** Audrey David, Ali Tahrioui, Anne-Sophie Tareau, Adrien Forge, Mathieu Gonzalez, Emeline Bouffartigues, Olivier Lesouhaitier, Sylvie Chevalier

**Affiliations:** Univ Rouen Normandie, Univ Caen Normandie, Normandie Univ, CBSA UR 4312, F-76000 Rouen, France

**Keywords:** *Pseudomonas aeruginosa*, biofilm, mechanical constraints

## Abstract

*Pseudomonas aeruginosa* is an opportunistic pathogen causing acute and chronic infections, especially in immunocompromised patients. Its remarkable adaptability and resistance to various antimicrobial treatments make it difficult to eradicate. Its persistence is enabled by its ability to form a biofilm. Biofilm is a community of sessile micro-organisms in a self-produced extracellular matrix, which forms a scaffold facilitating cohesion, cell attachment, and micro- and macro-colony formation. This lifestyle provides protection against environmental stresses, the immune system, and antimicrobial treatments, and confers the capacity for colonization and long-term persistence, often characterizing chronic infections. In this review, we retrace the events of the life cycle of *P. aeruginosa* biofilm, from surface perception/contact to cell spreading. We focus on the importance of extracellular appendages, mechanical constraints, and the kinetics of matrix component production in each step of the biofilm life cycle.

## 1. Introduction

Bacteria are able to adopt free-living or sessile lifestyles, leading pathogenic bacteria to cause acute or chronic infections, respectively, which are relevant for their survival and persistence in various environments. The free-living or planktonic lifestyle has been widely studied because growth conditions can be easily monitored in the laboratory. However, the sessile lifestyle, the so-called biofilm, is the most common form of organization found in ecosystems. It is defined as a three-dimensional multicellular complex of microorganisms aggregated within a self-produced extracellular matrix composed of exopolysaccharides (EPSs), proteins, lipids, nucleic acids, outer membrane vesicles (OMVs), and other minor components [1]. The matrix enables cell cohesion, surface attachment, and communication between cells. In addition, it allows for biofilm scaffold formation and protection from external aggressions, including antibiotics and immune system-related effectors [2]. While most of the laboratory studies focus on single-species biofilms, the large majority of environmental biofilms, however, are composed of various microorganisms (bacteria, fungi, archaea, viruses, etc.), leading to a highly complex community in which these microorganisms are in constant interactions [3]. Three types of biofilms have been described according to their structure and organization: (i) surface-associated biofilms, (ii) pellicles, and (iii) aggregates.

(i) Biofilms can develop on biotic surfaces, which are characterized by their biological origin such as living tissues, or onto abiotic nonorganic surfaces like metal, glass, plastic, or other synthetic materials. While abiotic-surface biofilms are often dominated by bacteria, biotic-surface biofilms seem to be more diverse in terms of microorganisms. Surface-associated biofilms are found in numerous environments, including water distribution systems, industrial cooling systems, and wastewater treatment equipment, where they are the leading causes of tube obstruction, material corrosion, and equipment deterioration. They are also associated with hospital-acquired infections, particularly those related to medical devices. These biofilms are described to be more resistant and more difficult to remove than other types of biofilms [2,4]. While numerous surface-associated biofilms have been described for their deleterious effects, they are also, and probably for most of them, beneficial, such as in symbiotic organisms like lichens and corals, which require biofilms for their survival and growth [5].
(ii) The second type of biofilms are the so-called pellicles that develop at the air–liquid interface. They are commonly found in aquatic environments such as rivers and lakes forming flocs, but also in fountains and faucets, where they can cause obstructions and infections. They are generally thinner, less structured, and more mobile than surface-associated biofilms since bacteria within pellicles can easily detach and spread into the environment [6,7]. Such pellicles play major functions in contaminant degradation and biogeochemical cycle regulation [8]. They can cause infections when they are inhaled or through contaminated medical devices [9]. In the food industry, they are commonly used to produce beer, yogurt, or kombucha [10]. Lastly, bacteria can auto-aggregate or co-aggregate.
(iii) Aggregates consist of a community in which bacteria bind to each other by physical interactions, such as cohesion, gravity, or turbulence, and chemical interactions with extracellular matrix exopolymers [11,12], without being attached to a surface or a pellicle. Aggregating biofilms are found in nutrient-rich environments, such as sediments and sludges. In hospitals, they can cause chronic infections, like in the lungs of cystic fibrosis (CF)-suffering patients, in the bladder leading to urinary tract chronic infections, or even on the skin, where they provoke chronic wound infections [13].

A characteristic aspect of sessile bacteria, which however remains poorly described, concerns the mechanical constraints they undergo during the different stages of biofilm formation and dispersion. These forces, however, can condition their behavior, and the biofilm lifestyle. In this review, we retrace the events of the life cycle of *P. aeruginosa* biofilm, focusing especially on the importance of mechanical forces, extracellular appendages, and the kinetics of matrix component production during this process.

## 2. The Life Cycle of *Pseudomonas aeruginosa* Biofilm

**Figure 1:**
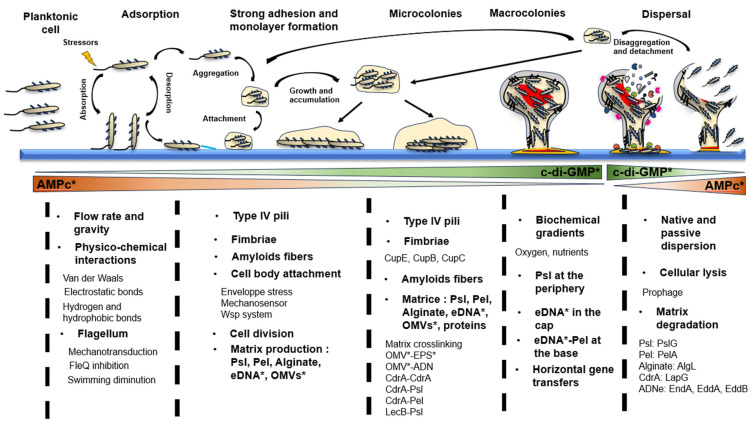
*P. aeruginosa biofilm life cycle.* The formation of a surface-associated biofilm is characterized by an initial stage of reversible adhesion. It depends on the flow rate of the surrounding fluid, gravity, and physico-chemical interactions. The bacterium attaches vertically by its polar flagellum. This attachment triggers a mechanotransduction process resulting in the activation of diguanlylate cyclases (DGCs), an increase in the intracellular bis-(3’-5’)-cyclic dimeric guanosine monophosphate (c-di-GMP) pool, and the inhibition of the positive regulator of flagellar genes, FleQ. The second stage involves strong adhesion and the formation of a monolayer by cell division on the surface. It engages various extracellular appendages (type IV pili, fimbriae, and amyloid fibers). The transition to a horizontal state results in the fixation of the cell body to the surface. Initial matrix components are produced, stabilizing the biofilm. Cell aggregates formed beforehand can also attach themselves directly to the surface, starting the process of forming microcolonies. The activation of other DGCs contributes to an increase in the c-di-GMP pool. The third stage involves the formation of microcolonies, facilitated by extracellular appendages that allow cell clustering. Production of matrix components continues, leading to significant cross-linking and strengthening of the overall biofilm structure. The final stage of biofilm maturation involves the formation of macrocolonies. The development of these mushroom-shaped structures leads to a rearrangement of the extracellular matrix and promotes horizontal gene transfer. Significant gradients of nutrients and oxygen are present within these structures. The dispersion occurs in response to various signals (nutritional, significant oxidative stress) leading to cell lysis in the upper part of the cap and the release of enzymes degrading the matrix. Cell detachment takes place in planktonic form or in aggregates that can join a surface or a biofilm in formation. The c-di-GMP levels decrease following the activation of different phosphodiesterases (PDEs). * c-di-GMP: bis-(3′-5′)-cyclic dimeric guanosine monophosphate; AMPc: cyclic adenosine monophosphate; eDNA: extracellular DNA; OMVs: outer membrane vesicles; EPS: exopolysaccharides.

The cycle of a biofilm is classically described as a five-stage process: adsorption, adhesion and monolayer formation, microcolonies formation, maturation, and cell dispersion to colonize a new surface. However, the type of biofilm formed is largely dependent on the environmental conditions encountered. More recently, a three-step model has been suggested that includes an aggregation (with or without surface attachment), a growth, and a disaggregation step, including the formation of biofilm in the form of pellicles or aggregates [13] (Figure 1). Each step is finely regulated and corresponds to unique gene expression and protein production patterns. These two models appear closely linked. It has been shown that bacterial aggregates, whether or not they are detached from the surface-associated biofilm, can become associated with a new surface or another type of biofilm that has already formed [14] (Figure 1). As the three-step model can be included within the first established model, we describe here the different steps constituting biofilm formation, from attachment to a surface to dispersion, to establish the life cycle of *P. aeruginosa* biofilm as completely as possible. Bacterial responses and biofilm formation are specific to the type of surfaces encountered [15,16], but general mechanisms can be described. 

### 2.1. Step 1: Adsorption

***Dynamic condition and mechanical constraints.*** Most of the studies from the literature describe biofilm formation in static conditions. However, it insufficiently mimics the environmental conditions. Indeed, biofilms are frequently observed in fluid flow conditions where pH, nutrient availability, salinity, physical transport, and hydrodynamic shear forces have a significant impact on the formation and metabolism of biofilm [17]. In fluid flow conditions, the kinetics of the fluid carrying the bacteria play a crucial role. At high flow, shear forces increase and limit the contact between bacteria and the surface. This mechanism generates a rotational movement of the elements near the surface, causing the weakly adhered bacteria to roll away. Swimming bacteria are trapped by their flagella in the rotation field [18]. Bacteria attached under identical flow conditions are more susceptible to detach when shear forces suddenly decrease, showing that individual cells respond to flow variations by modifying their adhesion state [19,20]. Cell sedimentation flow can counteract the ambient flow in some conditions and promote biofilm formation on horizontal surfaces. The influence of gravity on bacterial transport, adhesion, and biofilm formation is important in nature since *P. aeruginosa* biofilms formed under microgravity show different structures compared to those developed under Earth’s gravity conditions [21].

***Physicochemical interactions.*** In association with physical events, chemical interactions lead to bacterial surface attachment. The microenvironment near the surface differs from the liquid containing the bacteria. Differences in ionic strength, osmolarity, pH, and nutrient availability between liquid and surface influence the physicochemical interactions between bacteria and surface [16]. These interactions include Van der Waals forces, electrostatic, hydrophobic, and hydrogen interactions. They depend on the bacterial surface, the attachment surface, and the medium [22]. Nearing a surface, a bacterium is initially attracted by long-range Van der Waals forces. These forces increase as the distance to the surface decreases. Next, short-range electrostatic and hydrophobic forces are established and depend on the type of surface. For example, on anionic, unlike cationic surfaces, Gram-negative bacteria must resist the electrostatic repulsion caused by the negative charges of the surface-exposed lipopolysaccharides (LPSs) anchored into the outer membrane. These interactions involve hydrogen bonds, specific surface receptors, and extracellular appendages such as flagella [16].

***Appendages: a major role for the flagellum.*** Flagella are long, helical, and rotating appendages used by bacteria to move into liquid media or on semi-solid surfaces by swimming motility, with a speed exceeding several cell body lengths per second [23]. In *P. aeruginosa*, flagellar-mediated motility is required for biofilm formation, since bacteria with a functional flagellum can adhere by 100 times more efficiently during the early adhesion phase (30 min) [22]. In *Escherichia coli*, flagella have been shown to explore surface topology to increase adhesion in suitable regions [24]. Flagella crosslink the bacterial pole vertically to the surface [25,26], and the surface “pits” or “canyons” restrict flagellar rotation, leading to envelope deformation, and providing a mechanical signal [27]. Signal transduction is triggered following a reduced flagellar rotation resulting from the surface contact, or increased medium viscosity, as is the case of cystic fibrosis (CF) patients’ lungs. First described in *Bacillus subtilis,* this mechanical response was shown to be related to the DegS/U two-component system (TCS). The DegS sensor detects the flagellum rotation restriction, resulting in DegU response regulator phosphorylation, which in turn activates the transcription of genes encoding biofilm matrix components biosynthesis [28,29]. In *Vibrio cholerae*, alteration of flagellar rotation by contact with the surface also promotes bacterial adhesion [30]. 

Production of the flagellum in *P. aeruginosa* requires transcription of a large number of genes [31] and most of them are controlled by the transcriptional activator FleQ. At the same time, FleQ represses the expression of *pel* and *psl* operons, which encode major matrix exopolysaccharides (EPSs) [28,32]. FleQ activity is inhibited by intracellular levels of c-di-GMP [33]. By contrast to a planktonic lifestyle, a high level of c-di-GMP has been correlated to the biofilm lifestyle (Figure 1). This messenger is produced by DGCs and degraded by PDEs [34]. The actions of DGCs, such as GcbA or SadC, and PDEs, like BifA or DipA, were shown to affect swimming motility [35,36,37,38]. Similar to c-di-GMP, the HtpB phosphorelay is also involved in flagellar gene expression, and in the planktonic to sessile lifestyle switch. Phosphorylation of HtpB induces HsbR phosphorylation and HsbA dephosphorylation. As a result, the sigma factor FliA is released and it then binds to the RNA polymerase and initiates flagellar gene transcription. In the inactive state, HsbA is phosphorylated via HsbR and activates HsbD DGC which results in c-di-GMP production and type IV pili activity, thus orienting the bacteria into a biofilm lifestyle [39]. Altogether, these data suggest that surface contact perception and transduction result in an increased c-di-GMP pool and decreased flagellar gene expression and activity, promoting irreversible adhesion, notably via type IV pili activity.

### 2.2. Step 2: Strong Surface Adhesion and Monolayer Formation

In most cases, the absorption step is described as unstable. However, according to the environmental conditions and the perceived signals, adhesion may become irreversible, constituting the second step in biofilm formation. This approximately 2 h step requires the involvement of extracellular appendages such as type IV pili, fimbriae, and amyloid fibers, as well as increased adhesion forces. Bacteria change their orientation with regard to the surface, moving from a polar to a flat attachment of their body to explore the surface. At this step, bacteria develop into a monolayer by cell division (Figure 1) [14]. At this stage, activation of the Gac/Rsm pathway plays a major role in regulating bacterial cell adhesion in *P. aeruginosa*. Activation of the TCS GacAS leads to transcription of the small RNA (sRNA) *rsmZ* and *rmsY*. These sRNAs enable *psl* and *pel* transcription by sequestering the post-transcriptional regulator RsmA. Strong expression of *rsmY* and *rsmZ* favors initial attachment to surfaces and impedes subsequent biofilm development [40,41]. 

#### 2.2.1. Involvement of Appendages

**Figure 2:**
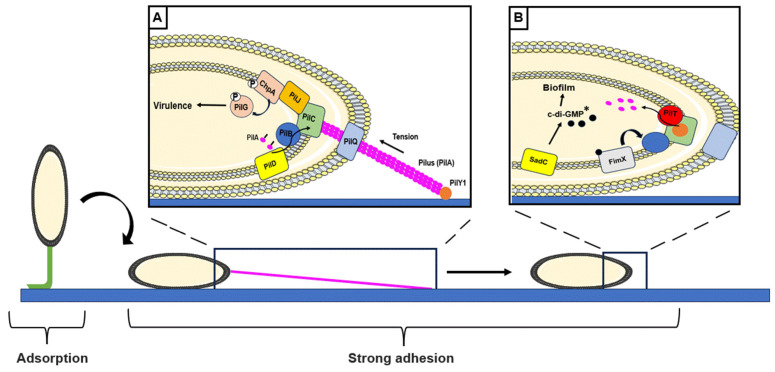
Type IV pilus contact with a surface in *P. aeruginosa*. The type IV pilus is composed of a pilus (PilA subunit) (pink) topped with the adhesin PilY1 (orange). The polymerization of PilA subunits is facilitated by PilC (green) after cleavage of the prepilin by PilD. The pilus emerges through the secretin PilQ (light blue). Elongation is powered by the ATPase PilB (blue) (**A**). Contact of PilY1 with the surface causes an interaction between PilA and PilJ sensors (yellow). This interaction leads to the phosphorylation of ChpA and PilG (PilG/ChpA TCS). This mechanoresponse leads to the production of virulence factors (**A**). Upon contact with the surface, depolymerization of the pilus is mediated by the ATPase PilT. PilY1 is in the inner membrane and activates the SadC diguanlylate cyclase (DGC). Production of bis-(3′-5′)-cyclic dimeric guanosine monophosphate (c-di-GMP) increases and its fixation on FimX leads to an increase in the activity of type IV pili (**B**). * c-di-GMP: bis-(3′-5′)-cyclic dimeric guanosine monophosphate.

***Type IV pili.*** Type IV pili are long retractable helical filaments that elongate by polymerization, adhere to a substrate, and retract by depolymerization providing the force to pull bacteria from the surface, leading to the flagellum-independent twitching motility. Type IV pili are also involved in the flagellum-dependent swarming motility that is based on the rapid and coordinated movement of a bacterial population on a semi-solid surface [42]. Type IV pili exercise a complementary surface attachment mechanism to that mediated by flagella. Contact between the pilus and the surface generates a mechanical tension along the pilus, which is transmitted to the cell via a mechano-transmission process. *P. aeruginosa* is able to detect stiffened surfaces by a mechanism based on the rate of diffusion of the pilin subunit PilA into the inner membrane during pilus retractation. This process leads to bacterial surface deformation, the intensity of which depends on the level of surface stiffness [43]. Besides its contact with the surface, the adhesin PilY1 that is located at the top of the pilus changes its conformation, promoting signal propagation (Figure 2A). Mutation of the human mechanosensory glycoprotein homologous PilY1 N-terminal domain reduces adhesion forces, c-di-GMP levels, and biofilm formation [44]. In fluids, it has been shown that PilY1 is able to detect shear forces. This mechanosensing process results in increased surface attachment and intracellular c-di-GMP levels [45]. After PilY1 attachment to a surface, signal propagation leads to activation of the PilG/ChpA TCS. The membrane sensor PilJ, when it interacts with PilA, induces the ChpA sensor followed by PilG response regulator phosphorylation (Figure 2A). This regulatory cascade results in increased intracellular cAMP levels and virulence factor production (Figure 2A) [29]. However, the mechanism of signal transmission between type IV pilus and Chp system remains unclear [46]. A recent study showed the involvement of PilT ATPase in surface detection and cAMP production and suggested a model in which PilT, but not PilA, would interact with PilJ [47]. *P. aeruginosa* rapidly activates PilT during surface contact, supporting its role in surface sensing [48]. During pilus retraction, PilY1 is localized at the inner membrane and activates the membrane DGC SadC. SadC activation increases the c-di-GMP pool and promotes biofilm formation (Figure 2B) [49]. The Pil-Chp system largely controls surface attachment in the *P. aeruginosa* PA14 strain. The *P. aeruginosa* PAO1 strain can attach faster than PA14 due to the involvement of the Wsp system (to be mentioned later within the section discussing cell body attachment) [50]. The c-di-GMP binding protein FimX was shown to interact with PilB and positively regulate type IV pilus activity [51] (Figure 2B). 

***The role of fimbriae.*** Fimbriae form filamentous structures that extend from the cell surface to anchor to substrates [52]. These structures are frequently reported to be involved in bacterial pathogenesis, facilitating bacterial attachment to host tissue and biofilm formation [53]. There are different types of fimbriae systems, which are generally composed of at least three compounds, including the pilin subunit, a chaperone, and an usher protein (CupA to CupE systems). The variety of these systems reflects the ability of *P. aeruginosa* to colonize numerous surfaces [54]. Mutants lacking the CupA system are impaired in biofilm formation. CupB and CupC systems are not involved in biofilm initiation [55]. By contrast, increased expression of the *cupD* operon leads to increased biofilm formation and reduced motility [56]. Finally, E-type fimbriae adopt variable curvatures in response to their environment, which could facilitate cell attachment [54].

***The role of amyloid fibers.*** Amyloid fibers are long and thin structures composed of FapC subunits. These subunits are organized in β-sheets, form fibril polymers, and extend from the outer membrane. These structures are intensively studied notably in neurodegenerative human diseases, such as Alzheimer’s disease, and exhibit structural homologies with bacterial amyloid fibers [57]. In bacteria, they are involved in biofilm formation, adhesion, host-cell invasion, and host–pathogen interactions [58], and an adhesin role was suggested in *P. aeruginosa*. In addition, purified fibril polymers adhere strongly to plastic and glass surfaces. The presence of amyloid fibers is not essential for biofilm formation, since a *fap* operon deletion mutant still displays the ability to form a biofilm in vitro [59]. By contrast, the *fap* operon is essential for the establishment of lung infection in rats. The expression of these operons correlates with increased production of surface mechanosensing proteins [60], and amyloid fibers affect the hydrophobicity and mechanical properties of *P. aeruginosa* biofilm [57].

#### 2.2.2. Importance of Cell Attachment

**Figure 3:**
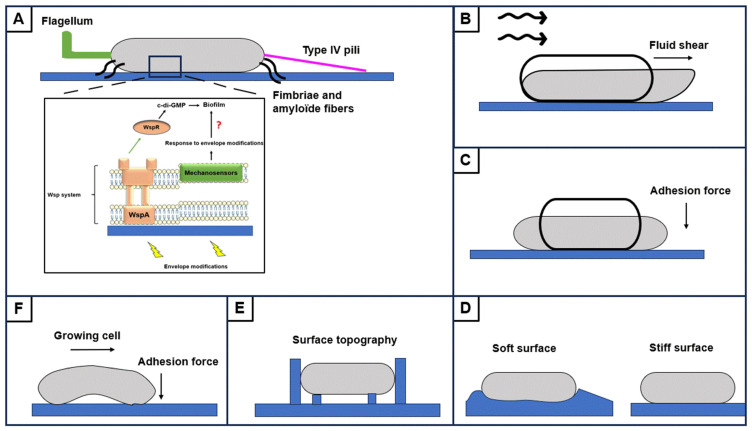
Mechanical forces deform the cell wall during cell attachment. Flagellum, type IV pili, and adhesins (fimbriae and amyloid fibers) are involved in cell attachment and prevent detachment (**A**). Ambient flow generates shearing force and load to envelope deformation (**B**). Adhesion forces can lead to envelope deformation (**C**). Substrate stiffness can lead to cell wall deformation (**D**). Surface topography can impact the envelope (**E**). Pressure due to adhesion forces applies mechanical force on the cell wall during cell growth (**F**). Cell body contact with the surface activates the Wsp system. Envelope modifications sensed by WspA increase the pool of c-di-GMP due to activation of the WspR DGC. Membrane mechanosensors are potentially involved in sensing envelope deformations by unknown mechanisms. The perception of envelope deformations leads to an adapted response which could be associated with biofilm formation (**A**).

During the strong adhesion step, bacteria are attached to the horizontal axis. The cell envelope is in direct contact with the surface and some membrane proteins may be involved in surface mechanosensing [27]. During attachment, bacteria can be exposed to various mechanical constraints, such as shear forces generated by ambient flow, surface topography, and surface stiffness, which can affect cell morphology, membrane stiffness, and mechanosensory protein conformation (Figure 3). Envelope deformations create mechanical stimuli that are detected by a mechano-sensitive envelope protein, providing a mechanical signal converted into a biological signal, in the same way as the mechano-transduction mechanisms observed for flagella and type IV pili (Figure 3A) [27]. Forces generated by ambient flow, particularly shear forces, promote cell attachment. Increased adhesion occurs via extracellular appendages by independent mechanisms, suggesting the involvement of specific receptors that are sensitive to mechanical constraints [19,20]. One study identified *phoQ*, *mexS*, *glnk,* and PA5488 genes as specifically involved in *P. aeruginosa* adhesion under dynamic conditions. PhoQ is involved in the synthesis and modification of LPS, and MexS in the regulation of efflux pumps. Mutations in these genes lead to an attachment defect. GlnK is involved in the regulation of nitrogen metabolism and PA5488 encodes a protein recently identified as involved in the regulation of a phospholipase, which plays a role in membrane metabolism [61]. Their mutations lead to delayed surface attachment and biofilm formation [62]. Wsp surface detection system includes the WspA chemoreceptor, which recognizes a surface-associated signal and initiates a phosphorylation cascade leading to activation of the WspR DGC (Figure 3A). In *P. aeruginosa*, a recent study linked Wsp system to envelope modifications as a surface-associated signal. Activation of Wsp in case of envelope perturbations suggests that membrane tensions generated during attachment promote biofilm formation via the Wsp system [63]. A relationship between the inner membrane fluidity and WspR activity was then suggested [64]. In *P. aeruginosa*, membrane fluidity is regulated by the extra-cytoplasmic function sigma factor SigX that responds to envelope stress [65,66]. SigX regulates the expression of numerous genes including *oprF*, encoding the major outer membrane porin. OprF was shown to be involved in the response to polydimethylsiloxane stiffness [67]. In addition, SigX regulates *cmpX* expression, encoding a putative mechanosensitive ion channel [68]. These data suggest a relationship between the cell envelope stress response, the sensing of mechanical forces, and biofilm formation.

#### 2.2.3. Initiation of Extracellular Matrix Production

Biofilm formation is characterized by the production of a self-produced extracellular matrix that enables cohesion and communication between cells within the biofilm and forms a molecular sieve with varying pores from 500 to 100 nm limiting the penetration of smaller molecules [69]. Responsible for more than 90% of biofilm biomass, it provides a shelter for bacteria, protecting bacterial communities against immune system attacks and antimicrobial treatments [70]. In *P. aeruginosa*, the matrix is composed of water (97%), exopolysaccharides (EPS, 1–2%), nucleic acids (<1%), proteins, and outer membrane vesicles (OMVs, <1%) [71]. This composition varies according to the environmental conditions encountered as well as the biofilm maturity degree [72].

***Exopolysaccharides.*** Psl is a pentamer of D-mannose, L-rhamnose, and D-glucose linked by β-1,3 bonds. It can sometimes contain galactose and traces of xylose [73,74]. It is the main EPS of *P. aeruginosa* PAO1 that is not produced by PA14 because of a deletion of three genes of the *psl* operon [75]. When these two strains are in competition under dynamic conditions, the production of this EPS allows PAO1 to outcompete PA14 in the early stages of biofilm formation [50]. Thus, the two strains appear to have distinct surface association strategies but also differ in matrix composition. In PAO1, Psl is crucial for biofilm formation as it is necessary for surface attachment, microcolony formation, and maintenance [74,75]. When PAO1 moves along the surface, it deposits a ‘trail’ of this EPS serving as a molecular glue. This deposit promotes cell–surface and cell–cell contact by anchoring in a helical form on the cell surface [74,76]. Psl also enhances surface motility upstream of biofilm formation [77]. Pel is a cationic EPS composed of a dimeric repeat of galactosamine and N-acetylgalactosamine linked by α-1,4 bonds [78]. It is produced by PA14 and PAO1 strains. Essential for biofilm formation in PA14, the absence of Pel in PAO1 has no effect on biofilm formation. However, when PAO1 is deficient in Psl, a significant production of Pel counteracts the effects of Psl absence during biofilm formation [75,79]. This EPS was first identified in mutants deficient in pellicle formation [24]. Like in PAO1, Pel is required for the initiation and formation of microcolonies by promoting interactions within the biofilm and playing a scaffolding role [80]. However, Pel seems to be involved in surface attachment only under certain conditions, and PA14 appears more effective than PAO1 in colonizing preformed biofilms [50,81]. In PAO1, it enhances surface detection by increasing shear resulting from twitching motility [45]. Alginate is the third major type of EPS produced by *P. aeruginosa*. It is a high molecular weight acetylated polymer composed of non-repetitive monomers of L-guluronic and D-mannuronic acids linked by β-1,4 glycosidic bonds [82]. Alginate is produced by PAO1 and PA14, and is not essential for biofilm formation, unlike the other two EPS [83]. However, it is found in large quantities in mucoid strains that are frequently found in the lungs of CF-suffering patients. Alginate binds to the mucin present in the respiratory tract of patients and acts as an adhesin, initiating biofilm formation. In this context, the regulatory mechanisms of alginate production are extensively studied to identify new therapeutic targets. 

***Extracellular DNA (eDNA).*** Another major component of the matrix is eDNA. Its genomic and plasmidic origin has been demonstrated [84]. Its release occurs through a spontaneous and multifactorial process inducing cell lysis [85]. The release of eDNA in *P. aeruginosa* is regulated by quorum sensing (QS)-dependent and independent mechanisms. QS is a cell density-dependent regulatory mechanism. In *P. aeruginosa*, the Las and Rhl QS systems use N-acyl-homoserine lactones (AHLs), while the PQS system relies on 2-alkyl-4-quinolone (HAQ, PQS system) signaling autoinductors [86,87]. When the concentration of a signaling molecule reaches a critical threshold (quorum) it binds to the corresponding regulator and enables regulation of a large number of target genes [88,89]. QS-induced cell lysis mainly occurs upstream of biofilm formation, and the released eDNA subsequently contributes to biofilm stabilization. The PQS QS-system triggers eDNA release by inducing prophage expression that leads to cell lysis [84]. QS activity also enables the production of a virulence factor, pyocyanin, inducing cell lysis and promoting eDNA release [90]. Mutation of the gene encoding the heat shock protein DnaJ results in altered pyocyanin production, reduced biofilm formation, and eDNA release. This reduction has been attributed to the alteration of QS gene expression [91]. Once released, eDNA promotes surface adhesion by increasing the hydrophobicity of the cell envelope [92]. It also facilitates biofilm expansion induced by coordinating cell motility on the surface [93].

***Outer membrane vesicles (OMVs).*** Their biogenesis mechanism is not yet well-defined. It is assumed that OMVs are formed by the outward expansion of the outer leaflet of the outer membrane, creating a membrane bulge that attracts periplasmic content into the vesicle before detaching. The envelope is the first cellular element in contact with the environment. Thus, environmental changes directly impact the envelope and can lead to a significant production of OMVs. This is indeed the case during exposure to certain antibiotics or under osmotic stress conditions. OMVs formed by *P. aeruginosa* are found to be enriched in LPS from the originating outer membrane, which makes the initial cell surface more hydrophobic, facilitating adhesion and biofilm formation [94,95]. Vesicles formed by planktonic cells primarily contain virulence factors such as peptidoglycan degrading enzymes and proteases. These virulence factors affect the host epithelium or other bacteria already present on a surface to promote the establishment of the producing bacterium and increase the available nutrient source [96].

### 2.3. Step 3: Microcolonies Formation

Once the bacterial monolayer is formed and the initial matrix elements are produced, the emergence of immature structures called microcolonies is observed. These microcolonies are aggregates of approximately 50 cells embedded in the extracellular matrix (Figure 1). The forces applied during cell division, extracellular appendages, and EPS enable their formation. They serve as the foundation for more complex three-dimensional (3D) structures formed later on [13,76].

#### 2.3.1. Physical and Mechanical Constraints

The formation of microcolonies involves mechanical couplings between cell elongation forces during division, substrate adhesion forces, and vertical cellular rearrangement. During division, adhesion is asymmetrical, with the newly forming poles not yet adhered to the surface. This asymmetric distribution of adhesion forces on the envelope contributes to the shape of the microcolonies. Once the monolayer is formed, turgor pressure accumulates within the cells and becomes greater than adhesion forces. The forming pole then positions itself above the neighboring cell, initiating the process of vertical cellular rearrangement [97,98,99].

#### 2.3.2. Appendages

The classical model of microcolony formation involves the loss of flagellar motility through negative regulation of flagella-related genes and positive regulation of EPS production-related genes. This pattern aligns with the regulatory systems mentioned earlier, promoting the transition between planktonic and biofilm lifestyles, with the non-motile adhered bacteria becoming sessile [33,39]. However, data regarding the presence of flagella during the biofilm life cycle are still contradictory. Recently, a study demonstrated the presence of flagella throughout the biofilm maturation process, particularly in the lower layers, suggesting an important role of flagella during biofilm maturation [100]. The activity of type IV pili enables the gathering of cells into microcolonies from the monolayer. Mutants defective in these pili can adhere to a surface and form a monolayer but are unable to agglomerate [101]. Fimbriae play a crucial role in the formation of these primary structures. After the initial adhesion phases, the operon encoding the CupE system is highly expressed, and its absence promotes a relatively flat biofilm [102]. Similarly, mutations in the *cupB* and *cupC* operons lead to reduced cell aggregation. Disruption of the CupC system appears to contribute predominantly to the observation of this phenotype [53]. Regarding the involvement of amyloid fibers, overexpression of the *fap* operon shows rapid and strong aggregation of *P. aeruginosa* [103]. While each of these appendages seems to play a more or less important role in surface attachment, their concomitant presence is necessary for the formation of microcolonies, likely facilitating cell gathering.

#### 2.3.3. Role of Matrix Compounds

***Exopolysaccharides***. Contrary to the macrocolonies formed later on, Psl is uniformly distributed within the microcolonies [73]. This EPS enhances the cross-linking of the matrix, establishing a scaffold that facilitates microcolony formation. This scaffold acts as a filamentous structure to construct the initial architecture of the biofilm and support the enlargement of microcolonies [104]. The expression of *psl* results in an upward tilt of the cells on the surface, promoting the vertical growth of microcolonies [105]. The significant presence of Psl in the sputum of CF-suffering patients is associated with bacterial aggregation and tobramycin tolerance [106]. Furthermore, *psl* expression reduces neutrophil phagocytosis and oxidative response by limiting opsonization [107]. As for Pel, it imparts a viscous aspect to the matrix, promoting cell spread and laterally directing growth, contributing to the horizontal expansion of microcolonies [104].

***eDNA***. At this step, the mechanisms of eDNA release appear to be QS-independent. Cell lysis occurs through prophage induction [108] and via the BfmRS TCS [109]. Interestingly, eDNA release may be mediated by flagella and type IV pili, since a *fliM-pilA* double mutant is defective in eDNA release [84]. DNA binds to calcium, creating a mesh system that promotes cell adhesion and microcolony formation [110]. In 4-day-old biofilms, microcolonies grow in height and form stalks constituting the base of mushroom-shaped macrocolonies. eDNA is then found in the outer parts of these stalks, and appears to be necessary for cap formation since DNase treatment prevents the formation of the upper caps of macrocolonies [101].

***Outer membrane vesicles (OMVs)***. Increased biomass promotes the production of OMVs by the PQS system. Biofilms formed by a *pqsA* mutant produce fewer OMVs than the wild-type strain [111]. Induction of prophage through the SOS response favors OMV production. After lysis, membrane debris re-circularizes to form vesicles that have randomly captured the released content from lysed cells, including eDNA [108]. Biofilm-derived OMVs differ from those of planktonic cells. While planktonic OMVs predominantly contain virulence factors, biofilm OMVs are smaller but more frequently associated with DNA [112,113], promoting horizontal gene transfer within the biofilm [114]. Two different plasmids containing β-lactam resistance genes incorporated into OMVs were able to be transferred to *P. aeruginosa* sessile cells [115]. The production of PQS-type molecule-carrying OMVs also enhances biofilm growth and resistance [116]. As OMVs originate from the outer membrane of bacteria, they include various adhesins (CdrA, lectins) on their surface, promoting cell aggregation. It has also been suggested that OMVs may bind to EPS and eDNA, thus contributing to the architecture of the biofilm [117].

***Matrix proteins***. Few data are available on matricial proteins. A comparison between the matrix proteome and the proteome of sessile cells revealed the presence of 45 proteins specific to the matrix [112]. These proteins are mainly associated with OMVs or originated from cell lysis within the biofilm. The most described ones include CdrA and the LecA and LecB lectins. The expression level of *cdrA* which correlates with the c-di-GMP level, is often used as a reporter tool for the small messenger level [118]. CdrA is an adhesin anchored to the membrane through a two-partner secretion system encoded by the *cdrAB* operon. CdrA-CdrA and CdrA-Psl interactions promote cell aggregation and stabilize the biofilm architecture by contributing to matrix cross-linking [118]. Binding with Psl provides CdrA protection against proteolysis, notably by self-produced elastases, thereby preserving the integrity of the biofilm [119]. CdrA also promotes cell self-aggregation even in the absence of EPS, showing its importance in biofilm cohesion [118]. More recently, CdrA was found to bind to Pel as well [120]. Lectins are proteins located in the outer membrane, with LecA and LecB that bind to galactose and fucose from prokaryote and eukaryote surfaces. These adhesins facilitate adhesion to biotic surfaces such as epithelia and mucous membranes [70]. LecA is highly expressed in sessile cells, and its absence affects the biofilm architecture [121]. Similar to CdrA, LecB binds to Psl, promoting cell aggregation and matrix stabilization. LecB also coordinates the localization of Psl in the biofilm. Lectins are particularly important during biofilm maturation. When *P. aeruginosa* is cultured in the presence of monosaccharides binding to lectins, there is an inhibition of biofilm maturation [122]. In Toyofuku’s study and those of his colleagues, the majority of the remaining matrix proteins are outer membrane proteins found in OMVs [112].

### 2.4. Step 4: Macrocolonies Formation

#### 2.4.1. Architecture and Biochemical Gradients

Bacterial biomass continues to increase, and the microcolonies grow larger. A 3D structure is observed (Figure 1) [13,123], which is called the macrocolony or ‘mushroom’ due to its mushroom-like shape. Their presence is considered the final stage of biofilm maturation. It is important to note that this architecture is highly dependent on environmental conditions such as physico-chemical and biological factors, the speed and mechanical constraints due to external flow, as well as nutrient availability and adhesion surface [50,123]. The formation of macrocolonies is triggered by cell overcrowding and competition for nutrients within the microcolonies. These phenomena prompt bacteria to move upwards within the structure. It is generally accepted that the mushroom stalks are formed by the proliferation of a non-motile subpopulation, and the mushroom caps, by a population using type IV pili to climb the stalks to aggregate at its summits [101,124,125]. However, a study showed that constant flagella-dependent dispersion occurs within macrocolonies, and the presence of flagella provides mechanical and physical support throughout the biofilm lifecycle [100]. These macrocolonies are separated by water channels allowing the circulation of various fluids or gases such as oxygen or nutrients [126,127]. *P. aeruginosa* exhibits distinct physiological characteristics (structures and metabolic changes) at different stages of biofilm development. The mature biofilm is the stage that shows the most differences in terms of gene expression across biofilm regions. On the surface, cells display an active growth metabolism with high gene expression and significant access to nutrients and oxygen. In the deeper layers, resource access is restricted, and cells enter a state of ‘dormancy’ with lower metabolic activity. These metabolically inactive cells are called ‘persisters’ and exhibit high resistance to antimicrobials. Metabolic, nutritional, and oxygen gradients are thus present within macrocolonies [2,128]. In addition to the involvement of c-di-GMP and TCS, the QS plays a significant role in the formation of these 3D structures. With high bacterial density, this system is activated extensively. Mutations in one of the three systems, particularly the Las system, result in biofilms devoid of macrocolonies. Studies have shown that QS is not essential to biofilm formation but plays a crucial role in the maturation stage. QS is also involved in the expression of the *rhlAB* operon, allowing the synthesis of surfactants called rhamnolipids. This operon is highly expressed in the stalk regions. The production of these rhamnolipids is necessary for maintaining the macrocolonies and associated networks of channels [129]. The involvement of QS systems underscores the importance of coordinated bacterial communication during biofilm maturation [123].

#### 2.4.2. Influence of Mechanical Constraints on Biofilm Architecture

The transport of dissolved compounds within the biofilm matrix is governed by a diffusion gradient that plays a crucial role in the development of the biofilm, influencing nutrient, water, and oxygen concentrations. The physical structure of the biofilm (porosity, surface density, elasticity) affects its mechanical resistance to encounter flow constraints (tensile strength, compression), and determines its deformation, rupture, and detachment. The viscoelastic properties of the matrix rely mainly on the EPS and play a crucial role in macrocolony formation. This viscoelasticity promotes bacterial cells to adhere to the surface and to each other, facilitating the formation of cellular aggregates. It also enables nutrients and water to be trapped and distributed, promoting macrocolony growth. These properties also make macrocolonies highly resistant to mechanical stress, enabling them to deform and return to their initial state unaltered [130,131]. The study of biofilms under dynamic conditions is complex and depends on flow velocity and nutrient concentration, as well as on shear stresses. Flow velocity mainly influences the thickness and density of the biofilm, while shear stresses affect the biofilm architecture. A study was conducted on *Pseudomonas fluorescens* under three different flow conditions. Under stagnant conditions, the biofilm is porous and thick. In laminar flow conditions with low shear rates, the biofilm is organized in aggregates. However, when the shear rate is higher, biofilms are filamentous with denser and thicker structures, and a matrix primarily located at the base of the biofilm [132]. The majority of studies on biofilms show that an increase in flow velocity leads to the formation of smooth and compact biofilms [133]. Flow constraints also influence the matrix production and localization. EPS and protein abundance was shown to increase with regard to the flow velocity enhancement [132,134,135]. In *P. aeruginosa*, the flow velocity and the flow rate increase are also associated with biofilm formation [136,137], and overexpression of the mechanosensitive ion channel CmpX leads to increased biofilm formation and major changes in architecture [137]. High-shear flow conditions lead to the formation of a thick biofilm with mushroom-shaped structures, whereas low-shear laminar flow results in a flat biofilm with small microcolonies [138]. By contrast, strains isolated from the lungs of CF-suffering patients are unable to form a biofilm when shear stresses are high, a phenotype that could correspond to the viscous environment with low shear stresses that occurs within the patient’s lungs. Under these conditions, a positive regulation of genes involved in stress response, alginate biosynthesis, and maintenance of cell shape (*mreBCD*) has been observed, while genes involved in the biosynthesis of virulence factors and efflux systems are downregulated [139]. Moreover, hydrodynamic conditions have been shown to have more influence than QS on the biofilm architecture [140]. Shear stresses also have an effect on the action of antibiotics within the biofilm. Various studies have demonstrated increased resistance of biofilms to antibiotics when subjected to shear stresses [141,142]. Rheotaxis, i.e., the study of bacterial movement in response to the amplitude and orientation of shear gradients, and rheology, reflecting the ability of bacteria to withstand these stresses, are increasingly being investigated. These phenomena are related to the mechanotransduction capability of bacteria. In *P. aeruginosa*, the *fro* operon has been identified as rapidly and robustly upregulated in response to fluid flow. The increased expression of this operon occurs through the action of the extracytoplasmic function sigma factor FroR, for which no surface sensor has been identified yet [143].

#### 2.4.3. Matrix Production

***EPS***. The production of EPS begins during the strong adhesion and continues during biofilm maturation. However, there is significant variability among strains regarding the contribution of Pel and Psl to the mature biofilm structure. In PAO1, the expression of Psl is necessary for maintaining the architecture of the biofilm in advanced maturation stages. Overproduction of Psl is associated with large macrocolonies [144]. This EPS accumulates at the periphery of the macrocolonies, forming a protective capsule. Its absence in the biofilm deepness reinforces the concept of phenotypic heterogeneity within a biofilm [74]. As mentioned earlier, Pel is not required for biofilm formation in PAO1 and only presents structural redundancy with Psl. Nevertheless, a shift in the production of Psl to Pel seems to occur in the later stages of maturation. Pel would then contribute to increased cell “trapping”, contributing to the formation of larger and denser macrocolonies. A substantial presence of Psl is associated with significant cross-linking and rigidity of the biofilm. Its reduction could facilitate dispersion in the later stages of maturation and represent an important adaptation strategy for *P. aeruginosa* in dynamic and fluctuating environments [79,104]. Although their contribution is generally less significant than that of alginate, Pel and Psl also contribute to matrix viscosity. Interactions between Pel, Psl, and other matrix components also reinforce the mechanical stability of the biofilm [145]. The presence of alginate is not essential to biofilm formation in non-mucoid strains, but it still plays a role in the structural stability of mature biofilms and cell viability. Alginate is an EPS that imparts high viscosity to the biofilm matrix, increasing the biofilm’s resistance to deformation and shear forces [146]. A mutant deficient in alginate biosynthesis develops biofilms with a reduced proportion of viable cells in the cap, promoting early biofilm dispersion [79]. The overproduction of alginate protects *P. aeruginosa* from the challenging environment of the lungs in CF-suffering patients. It imparts viscosity to the matrix by influencing viscoelastic properties, allowing the retention of water and nutrients within the biofilm. This viscosity also leads to impaired clearance within the lungs of infected patients [147].

***eDNA***. A high concentration of eDNA is found in the cap [84]. This may also contribute to consolidating the biofilm structure [148]. eDNA has elastic properties and contributes to matrix viscosity by forming polymeric networks with other components, particularly with Pel and Psl [149]. For example, Psl can be inserted into the DNA double helix by forming hydrogen bonds [150]. These bindings form a matrix scaffold mainly found in the stalks and consolidate the entire matrix structure [150,151]. eDNA also traps nutrients by forming ionic bonds [3,152]. In addition to its role in adhesion and biofilm stability, eDNA also participates in horizontal gene transfer. This involves a natural transformation phenomenon where naked DNA from the environment is imported through a specialized transport apparatus. Here, the type IV pilus facilitates this process by pulling eDNA back into the cell upon retraction. This process enables the evolutionary adaptation of the bacterial population to its environment and the acquisition of antibiotic-resistance genes [114]. This is particularly true in the case of aminoglycosides in Gram-negative bacteria.

### 2.5. Step 5: Biofilm Dispersion

Once the mature biofilm is established, it can continue to thrive or initiate a cellular dispersion if the environmental conditions are not conducive to the biofilm. A restructuring of the biofilm structure takes place, allowing the release of dispersed cells or aggregates (Figure 1). These cells can then colonize a new environment, join other established biofilms, or recolonize the same site when conditions become favorable again [153]. Since dispersed cells and the remaining cells are more susceptible to antimicrobial agents, the dispersion phenomenon is extensively studied in anti-biofilm strategies [154].

#### 2.5.1. Native Dispersal

The formation of biofilm has been presented as an advantageous and protective structure for bacteria. Multicellularity, in which cells adhere to and communicate with each other, leads to the formation of physical and chemical gradients, due to physical constraints and endogenous metabolic activity. Such gradients determine various microenvironments within the biofilm, influence the biofilm architectural development, and promote subpopulation emergence [155]. These bacterial subpopulations are characterized by metabolic, physiologic, and genetic diversity, and respond to gradients of molecules. For example, bacteria enclosed in the biofilm encounter low levels of oxygen and nutrients, while bacteria from the biofilm surface may be in direct contact with some carbon sources and oxygen [155]. These multiple microenvironments lead to the generation of various bacterial responses, including the general stress response driven by the sigma factor RpoS. However, this adaptation is only possible in the presence of moderate gradients. When these gradients are too pronounced, cell dispersion occurs to allow bacteria to survive. This phenomenon occurs in response to signals or signaling molecules synthesized by resident cells within the biofilm [156]. For example, sessile cells initiate a denitrification process, releasing nitric oxide that causes the lysis of peripheral cells [157]. The availability of nutrients can also act as a signal for dispersal. For example, the depletion of glucose sources triggers the dispersion of the biofilm, allowing bacterial survival by colonizing a more favorable environment [158]. Inversely, a sudden increase in a glutamate source induces the breakdown of the biofilm. This dispersal has been linked to an expression upregulation of *fliC* and downregulation of *pilA*, a pattern observed with succinate and glucose as well [159]. The abundance of nutrient resources is perceived as a favorable condition for bacterial development, eliminating the need for protection within the biofilm. Host-produced molecules can also act as signals for dispersal. For example, the presence of the bile acid taurocholic acid induces cell lysis, releasing cells from the deeper layers [160,161]. Increased temperature, variations in medium pH, and the presence of heavy metals are also examples of signals [153,162,163]. The perception of these dispersal signals involves a protein complex including the membrane proteins NicD and NbdA/MucR, which activate the BdlA protein, which in turn recruits and activates the two PDEs RbdA and DipA, promoting the reduction of c-di-GMP levels, and the planktonic phenotype [156].

#### 2.5.2. Passive Dispersal

Passive dispersal refers to the direct removal of cells from the biofilm, independent of bacterial responses. It can occur throughout the biofilm life cycle and mainly involves three phenomena resulting from mechanical stresses. Abrasion occurs following the collision of particles with the biofilm. Elimination is due to the continuous shearing of a liquid over the biofilm, leading to the erosion of individual cells or aggregates. Desquamation is the periodic release of biofilm clumps, independently of fluid shear [163]. These modes of removal are the result of physical and mechanical disturbances. For example, a sudden increase in flow rate increases shear forces and leads to the removal of part of the biofilm. Another example is the use of a toothbrush, which allows for the abrasion of the biofilm in dental plaque [164].

#### 2.5.3. Restructuring the Biofilm Architecture

**Figure 4:**
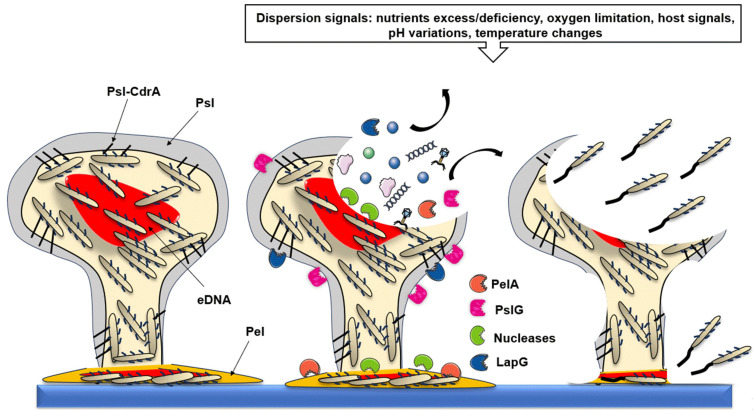
Biofilm dispersion. Biofilm dispersion is induced in response to the perception of a wide range of signals, such as nutrient excess or deficiency and significant pH or temperature variation, for example. The detection of these signals leads to cell lysis in the upper part of the macrocolonies. This lysis is triggered by the induction of prophages by QS systems (PQS and Rhl, mainly). The released cellular content contains numerous proteases: PslG degrades Psl in the periphery and enables the degradation of CdrA by LapG. PelA degrades Pel at the base of macrocolonies. Nucleases alter the eDNA located in the upper part and at the base of the stalks. Cells from the deep layers regain flagellar mobility and disperse. The degradation of the stalk reduces bacterial contact with the surface, allowing them to disperse.

The perception of dispersal signals leads to a reorganization of the biofilm, resulting in its breakdown. Significant cell lysis events occur in the upper part of the macrocolonies, forming a central cavity filled with matrix and debris. These debris include enzymes involved in matrix degradation [74]. For instance, Psl, located at the periphery of the macrocolonies, is degraded by the glycoside hydrolase PslG. Pel, mainly located at the base of the stalks, is degraded by PelA hydrolase. Alginate is degraded by the alginate lyase AlgL [165,166]. The adhesin CdrA, when not protected via its binding to Psl, is degraded by the LapG protease. Finally, eDNA is degraded by the EndA, EddA, and EddB nucleases (Figure 4) [166]. In addition to enzymes involved in matrix degradation, biofilm dispersion is facilitated by the production of rhamnolipids. These surfactants reduce surface tensions between cells and the surface and/or between two cells, facilitating disaggregation [153]. With the lysed cells at the periphery and the degraded matrix, viable cells from the deepest layers are then exposed at the surface of the formed cavity and can disperse following the induction of genes responsible for flagellum synthesis.

## 3. Biofilm Treatment

### 3.1. Antibiotic Resistance and Tolerance

Biofilm cells can be up to 1000 times less sensitive to antimicrobial agents than planktonic cells [167], causing a major problem in the treatment of biofilms. Resistance reflects the cell’s ability to divide in the presence of high antibiotic concentrations. Part of this resistance involves the modulation of gene expression. In *P. aeruginosa*, increased intracellular levels of c-di-GMP during biofilm formation correlate with increased expression of genes involved in antibiotic resistance [168]. In addition, cells within a biofilm show a high mutation rate, and the presence of antibiotics amplifies the oxidative stress present within biofilms [169]. All these phenomena favor the appearance of mutations aimed at selecting the best-adapted cells. A high frequency of horizontal gene transfer within biofilms has also been observed and is largely responsible for the acquisition of antibiotic resistance genes [170]. In addition, biofilm formation enables persistence and tolerance to a wide range of antibiotics. Tolerance is defined as the cell’s ability to survive in the presence of inhibitory antibiotic concentrations. This phenomenon has been increasingly studied in recent years, not least because it has been shown to facilitate the evolution of resistance [171]. In *P. aeruginosa*, this phenomenon is mainly attributed to the extracellular matrix that limits the penetration of antibiotics into the biofilm, the physiological heterogeneity of cells within biofilms, and the presence of so-called “persisters” [145,172,173,174,175]. As mentioned above, populations located at the biofilm periphery display high physiological activity, while sub-populations located in the inner parts display low activity or no growth (persisters), affecting the efficacy of all bactericidal antibiotics [14,176].

### 3.2. New Antibiotics

Thus, with the emergence of multi-resistant bacteria and persistent cells, conventional antibiotics have lost their initial efficacy. However, research and development of new antibiotics remain an important avenue for combating biofilm infections (for a recent review, see [177]). Recently, a new synthetic retinoid antibiotic (CD437), in synergy with gentamicin, has been shown to eliminate persistent biofilm cells formed by methicillin-resistant *S. aureus* (MRSA). Similarly, a new antibiotic (V-r8) combined with vancomycin and a guanidine-rich transport protein effectively eliminated (97%) MRSA biofilms and associated persistent cells, by destroying the bacterial membrane. Pentobra is a new antibiotic obtained by inserting 12 amino sequences encoding various antimicrobial peptides into tobramycin. Improving membrane permeability has proved effective against biofilms formed by *E. coli* and *S. aureus* [178]. Nitric oxide has been shown to be effective in dispersing biofilm. However, this molecule is difficult to manipulate and its delivery to a target site is complex. Thus, a nitric oxide-releasing antibiotic was synthesized. The nitric oxide donor diazeniumdiolate was covalently linked to cefaloram. In *P. aeruginosa*, this molecule, used at a concentration of 10 µM, eliminated almost 70% of the cells residing in the biofilm [179]. On the other hand, nitroxides, which are more stable than nitric oxide, have shown anti-biofilm properties. This led to the development of nitroxide-functionalized antibiotics. For example, ciprofloxacin-nitroxide-10 (40 µm) eradicated 95% of biofilm-resident cells in *P. aeruginosa*. Ciprofloxacin-nitroxide-27 (64 µM) also eliminated 99% of the biofilm formed by *S. aureus* [179,180]. Another study has studied the effect of treatment at high concentrations with aztreonam and tobramycin on *P. aeruginosa* biofilms showing their effect on filamentation, flattening, or disruption of the biofilm structures and their ability to reduce bacterial load and biomass [181]. Emerging promising combinations of inhaled antibiotics such as clarithromycin/tobramycin or colistin/tobramycin are still at a very early stage of development [182]. 

## 4. Concluding Remarks and Future Directions

Thus, the eradication of biofilm with 100% effectiveness through the use of antibiotics remains complicated. The residual cells can then form a new biofilm when conditions are favorable again. Similarly, the concentration of the antibiotics used must be perfectly controlled. Indeed, biofilm formation responds to a variety of environmental signals including the presence of sub-inhibitory concentrations (sub-MIC). These concentrations are defined as being below the minimum concentration necessary to inhibit bacterial growth (MIC). Bacteria are often exposed to intermittent or low concentrations of antibiotics. For example, this is the case in humans when there are errors in drug dosage, non-compliance with prescriptions, or in the case of bacterial development in a highly compartmentalized bronchial environment, leading to a gradient in the spread of the antibiotic. Cells within a biofilm are also exposed to a lower concentration of antibiotics than cells in the periphery. This phenomenon is largely involved in antibiotic tolerance and accelerates the emergence and spread of resistant bacteria [183], and in several common clinical pathogens such as *S. aureus*, *Enterococcus faecalis*, *E. coli,* and *P. aeruginosa*, exposure to sub-MIC concentrations of antibiotics induces biofilm formation [171,184,185,186,187,188]. It is important to better understand the structuring of biofilms over time in order to develop new control strategies. Thus, while few studies focus on the impact of mechanical constraints on biofilm formation, we point out through this review that fluid flow and contact with surface or other cells are signals that influence biofilm formation and architecture. These constraints play significant roles in the biofilm initiation, maturation, and dispersion. In particular, phenomena resulting from the deformation of the cell envelope, during contact of the cell or extracellular appendages with surfaces or other entities (cells, particles in the environment, shear forces), initiate a signaling cascade leading to the production of extracellular matrix components. The envelope is in direct contact with the environment and includes a number of mechanosensitive membrane proteins able to sense deformations of the wall structure. The perception and transduction of these mechanical signals lead to an adaptive physiological response, influencing stages of biofilm formation as well as its architecture. Furthermore, a significant number of regulators involved in membrane homeostasis such as AlgU or SigX are also implicated in biofilm formation. Thus, the study of the regulatory mechanisms involved in cell envelope homeostasis could provide a better understanding of the phenomena underlying biofilm formation and establish new therapeutic strategies.
